# Improved RNA–DNA interaction calling suggests RNA-based gene regulation of phenotypic transitions

**DOI:** 10.1093/nar/gkag304

**Published:** 2026-06-08

**Authors:** Simonida Zehr, Sandra Seredinski, Katalin Pálfi, James A Oo, Emma C Walsh, Alessandro Bonetti, Matthias S Leisegang, Ralf P Brandes, Marcel H Schulz, Timothy Warwick

**Affiliations:** Goethe University Frankfurt, Institute for Cardiovascular Physiology, Theodor-Stern-Kai 7, 60590Frankfurt am Main, Hesse, Germany; German Center for Cardiovascular Research (DZHK), Partner site Rhein-Main, 60590 Frankfurt am Main, Germany; Goethe University Frankfurt, Institute for Cardiovascular Physiology, Theodor-Stern-Kai 7, 60590Frankfurt am Main, Hesse, Germany; German Center for Cardiovascular Research (DZHK), Partner site Rhein-Main, 60590 Frankfurt am Main, Germany; Goethe University Frankfurt, Institute for Cardiovascular Physiology, Theodor-Stern-Kai 7, 60590Frankfurt am Main, Hesse, Germany; German Center for Cardiovascular Research (DZHK), Partner site Rhein-Main, 60590 Frankfurt am Main, Germany; Goethe University Frankfurt, Institute for Cardiovascular Physiology, Theodor-Stern-Kai 7, 60590Frankfurt am Main, Hesse, Germany; German Center for Cardiovascular Research (DZHK), Partner site Rhein-Main, 60590 Frankfurt am Main, Germany; Department of Biochemistry, University of Cambridge, Cambridge, CB2 1QW, United Kingdom; Discovery Sciences, Biopharmaceuticals R&D, AstraZeneca, Cambridge, CB2 0AA, United Kingdom; Goethe University Frankfurt, Institute for Cardiovascular Physiology, Theodor-Stern-Kai 7, 60590Frankfurt am Main, Hesse, Germany; German Center for Cardiovascular Research (DZHK), Partner site Rhein-Main, 60590 Frankfurt am Main, Germany; Goethe University Frankfurt, Institute for Cardiovascular Physiology, Theodor-Stern-Kai 7, 60590Frankfurt am Main, Hesse, Germany; German Center for Cardiovascular Research (DZHK), Partner site Rhein-Main, 60590 Frankfurt am Main, Germany; German Center for Cardiovascular Research (DZHK), Partner site Rhein-Main, 60590 Frankfurt am Main, Germany; Goethe University Frankfurt, Institute for Computational Genomic Medicine, Theodor-Stern-Kai 7, 60590 Frankfurt am Main, Hesse, Germany; Goethe University Frankfurt, Institute for Cardiovascular Physiology, Theodor-Stern-Kai 7, 60590Frankfurt am Main, Hesse, Germany; German Center for Cardiovascular Research (DZHK), Partner site Rhein-Main, 60590 Frankfurt am Main, Germany

## Abstract

Chromatin-localized RNAs play diverse roles in gene regulation and nuclear architecture. Mapping genome-wide RNA–DNA interactions is possible using a variety of molecular methods, including using bridging oligonucleotides to ligate RNA and DNA in proximity. While molecular methods have progressed, a robust computational method for calling biologically meaningful RNA–DNA interactions from these data is lacking. Herein, we present *RADIAnT*, a reads-to-interactions pipeline for analyzing RNA–DNA ligation data. *RADIAnT* calls interactions against a dataset-specific, unified background, which considers RNA binding site–TSS distance and genomic region bias, and outperforms previously proposed methods in the accurate recall of genome-wide RNA–DNA interactions. Accurate RNA–DNA interaction calling enables the analysis of gene regulatory RNAs in dynamic biological contexts. Here, dynamically chromatin-associated RNAs were identified in the physiologically- and pathologically relevant process of endothelial-to-mesenchymal transition. By depleting candidate chromatin-associated lncRNAs, their gene regulatory behavior at bound target genes important to endothelial phenotype maintenance could be validated. These data demonstrate how effective RNA–DNA interaction calling can help to place RNAs at key points in gene regulatory networks governing cellular behavior.

## Graphical Abstract

**Graphical Abstract:**
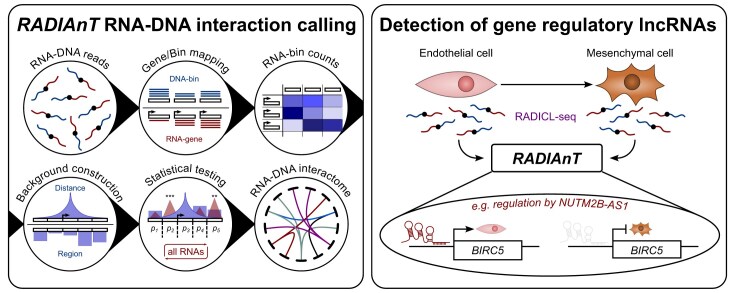

## Introduction

Where RNA was formerly viewed as a conduit between genes and proteins, it is now recognized as playing key roles across a vast number of cellular processes. Subcellular localization is a key regulator of RNA function [1], with functional RNA behavior being reported across almost every cellular compartment. However, some RNA transcripts never leave the nucleus [2] and remain associated with chromatin for their entire lifespan. Most notably, this has been shown for non-coding RNAs, such as *Xist*, whose role in X chromosome inactivation is crucial to mammalian development [3]. However, this is only one example from thousands of nuclear-localized non-coding RNAs, whose functions remain largely unknown.

Non-coding RNAs can be broadly classified into small non-coding RNAs (e.g. microRNAs, transfer RNAs, small nucleolar (sno)RNAs, and small nuclear (sn)RNAs) and long non-coding RNAs (lncRNAs), i.e. transcripts longer than 200 nucleotides [4]. LncRNAs generally show low evolutionary conservation and highly tissue- and cell type-specific expression patterns [5]. These differentiated profiles reflect the distinct functions lncRNAs perform across phenotypes, which, in turn, are often associated with a distinct mechanism of action of the given transcript in the given context. A lncRNA can act as a molecular scaffold for nuclear bodies, it can guide chromatin-modifying enzymes to targets sites, or it can inhibit an effector [6,7]. Nuclear-localized RNA molecules can also engage in direct interactions with genomic DNA, forming diverse molecular architectures, including R-loops, hybrid G-quadruplexes, and RNA–DNA triplexes [8]. Via the formation of these interactions, lncRNAs have been shown to modulate gene expression profiles, and thereby cellular behaviors [9]. A well-studied cell type in this regard is endothelial cells, where we and others have demonstrated the gene regulatory capacity of several nuclear-localized lncRNAs.

For example, *HIF1$\alpha$-AS1* forms triplexes at the loci of *EPHA2* and *ADM*, recruiting chromatin-modifying proteins to establish heterochromatin and thereby transcriptional repression [10]. *LINC00607*, which is highly expressed in endothelial cells, maintains endothelial identity by interacting with the chromatin remodeler BRG1 to maintain chromatin accessibility at target genes of the transcription factor ERG [11]. Similarly, *SMANTIS* interacts with BRG1 and modulates its genomic localization, preventing BRG1 binding at the *ICAM1* promoter and thereby limiting monocyte adhesion to endothelial cells [12, 13], a mechanism shown to be common to several other endothelial-expressed lncRNAs [14]. These findings, amongst others, illustrate the central role of lncRNAs in coordinating chromatin architecture and vascular gene regulation. This regulatory behavior is largely driven by accurate genome-wide localization of lncRNAs to regions of gene regulatory importance, a process which can be studied by several molecular methodologies.

One-to-all RNA–DNA interaction assays—such as ChIRP-seq [15], ChOP-seq [16], CHART-seq [17], RAP-DNA [18]—use biotinylated antisense oligonucleotides to capture genome-wide binding sites of a single RNA of interest. All-to-all methods, on the other hand, provide data on all RNA–DNA interactions in a given experiment. These methods include RADICL-seq [19], Red-C [20], GRID-seq [21], ChAR-seq [22], and iMARGI [23], all of which use bridging oligonucleotides that ligate RNA and DNA in spatial proximity, thereby detecting contacts between any transcript and any genomic locus. While one-to-all methods provide exact information about the target sites of an RNA of interest, all-to-all methods allow for the genome-wide and transcriptome-wide identification of chromatin-associated RNAs without prior selection of the RNA molecules involved.

Given the highly complex nature of all-to-all RNA–DNA ligation data, several computational methods have been proposed for their analysis. The RADICL-seq publication [19] suggests calling RNA–DNA interactions using a binomial test, comparing the mean RNA interaction count at a given genomic bin compared to the observed count. In GRID-seq [21], the authors propose building a background based on *trans*-chromosomal messenger RNA (mRNA)-DNA interaction frequencies, against which the fold enrichment of RNA–DNA interactions is calculated. This approach is also suggested by the authors of Red-C [20]. More recently, the peak-calling approach *BaRDIC* was published [24], which considers gene-interaction distance and variation in RNA-binding profiles to detect genome-wide RNA–DNA interactions from all-to-all RNA–DNA ligation data. Similarly to GRID-seq, *BaRDIC* utilizes mRNA–DNA contacts to build a statistical background. For interactions in *cis*, this background is additionally scaled by the distance between the RNA transcription locus and interacting region of the genome. *BaRDIC* calculates bin sizes and accounts for interaction-locus distance for each RNA individually, which results in long processing times and limitations in which RNAs can be analyzed, given the requirement for the presence of sufficient read counts to calculate these individual backgrounds. This means that, at present, there exists no standardized analysis for RNA–DNA ligation data that can effectively manage confounding factors such as distance and abundance, and eliminate experimental noise, while still maintaining sufficient sensitivity to detect biologically meaningful RNA–DNA interactions across a wide range of RNA abundances, all whilst being computationally efficient and user-friendly.

Here, we present *RADIAnT* (RNA And DNA Interactions Analyzed “n” Tested), a statistical method for calling robust interactions from diverse RNA–DNA ligation data types. By accounting for the main confounding factors in these data, *RADIAnT* is capable of detecting known RNA–DNA interactions with high specificity, whilst simultaneously remaining sensitive enough to identify interactions of lowly expressed RNAs. *RADIAnT* is incorporated into a complete reads-to-interactions pipeline, which can be implemented on any RNA–DNA ligation data with minimal pre-processing requirements. *RADIAnT* detects specific RNA–DNA interactions across different input data consistently, and outperforms other computational methods in accurate detection of genome-wide RNA–DNA interactions, whilst being more computationally efficient. This is demonstrated in an evaluation experiment utilizing orthogonal one-to-all RNA–DNA binding data across different species, ligation methods and RNAs. Given our experience in lncRNA-mediated gene regulation in endothelial cells, *RADIAnT* was implemented to detect dynamic gene regulatory RNA–DNA interactions from newly generated RADICL-seq data from the physiologically and pathologically relevant process of endothelial-to-mesenchymal transition (EndMT). Here, *RADIAnT* enabled the identification of putative process-specific regulators, whose gene regulatory effects on bound target genes were validated by lncRNA depletion. Thereby, we demonstrate that effective analysis of RNA–DNA ligation data enables the incorporation of RNAs into gene regulatory networks governing dynamic biological processes, such as phenotypic transitions.

## Materials and methods

### Identification of confounding factors

#### Identification of distance bias

RNA and DNA tags of mouse embryonic stem cells (mESCs) sequenced with GRID-seq (GEO accession no.: GSM2396700) and RADICL-seq (GEO accession no.: GSE132192) were mapped to the murine genome assembly version mm39 using *STAR* [25] (v2.7.3a) and annotated in GENCODE release M35. RNA and DNA tags of Red-C-sequenced human K562 cells (GEO accession no.: GSE136141) were mapped to the human genome assembly version hg38, annotated in GENCODE release 43. The distance between the tags was computed as the difference between the centre of the 5 kb bin the DNA tag was mapped to and either the start or the end (whichever lies closer) of the gene the RNA tag was mapped to. Gene start/end were rounded to 5 kb. We furthermore differentiated cases in which either (i) both tags mapped to the plus strand or (ii) both tags mapped to the minus strand (same-strandedness) from cases in which the tags mapped to different strands (opposite-strandedness), assuming that the latter would reflect nascent transcription. Visualization was produced with ggplot2 (v3.5.0).

#### Analysis of RNA abundance

To compare interaction frequency as captured by RADICL-seq, GRID-seq, and Red-C against gene expression, we mapped sequencing reads from CAGE (GEO accession no. GSE132191), GRO-sequencing (GEO accession no. GSE82312) and RNA-sequencing (GEO accession no. GSE136141), respectively, to the genomes as described in the section above using *STAR *[25] (v2.7.3a) and quantified using *featureCounts* (v2.0.0). Statistical analysis was conducted in R (v4.3.1), visualization with ggplot2 (v3.5.0).

### Preprocessing

#### Depletion of ribosomal RNA

Potential contaminating ribosomal RNA (rRNA) is eliminated with *BBDuk* of the *BBTools* suite (v39.19, https://github.com/bbushnell/BBTools). In our analysis of the data presented in this work, we used *BBTools* (v39.01) and the non-redundant SSU and LSU Ref datasets of the SILVA [26] (release 138.1) as a ribosomal reference database.

#### Mapping

The split RNA and DNA tags (FASTQ format) are aligned to the genome using *STAR *[25] (v2.7.3a). Apart from default parameters, we set –alignIntronMax 1 to disable splicing, –outFilterScoreMinOverLread 0 and –outFilterMatchNminOverLread 0 and –outFilterMatchNmin 0 to enhance the mapping rate. Multi-mapping DNA reads are discarded using *SAMtools* [27] (v1.10), whilst multimapping RNA read behavior can be determined by the user.

#### Identification of interacting regions

To describe interactions, the genome is split into two different kinds of units: For the DNA part of an interaction, bins of a fixed size (e.g. 5, 25, 50, 100 kb) are used. For the RNA part of an interaction, we regard genes as the smallest unit of the genome. *BEDtools* [28] *intersect* (v2.27.1) is used to assign aligned DNA tags to bins and RNA tags to genes, respectively. Since multiple genes can be annotated to the same locus, the read is assigned to the gene it has the maximum overlap with.

#### Distance computation

For intrachromosomal interactions, a linear interaction distance can be computed. Annotated gene starts and ends are rounded to match the center of the closest bin of a given size. The distance is then computed between either the rounded end or rounded start of the gene (whichever lies closer) and the center of the DNA bin it interacts with.

### Statistics

#### Background model construction

To account for experimental artifacts, we build a distance- and expression-sensitive background to model the expected interaction frequency of a given bin with a given RNA. The distance-specific background is calculated for each RNA $r$ as the number of reads that were sequenced at a given distance, normalized by the total number of reads for RNA $r$. For each given distance, the interaction frequency is given by the mean over all RNAs:


(1) \begin{eqnarray*}
\mathrm{ BG}_{\mathrm{ Distance}}(r, \mathrm{ dist}) = \frac{ \mathrm{ Count}(r, \mathrm{ dist})}{\sum _{b=1}^{n} \mathrm{ Count}(r, b)}\,\, ,
\end{eqnarray*}

where $n$ is the total number of bins on the chromosome where RNA $r$ resides. $\mathrm{ Count}(r, \mathrm{ dist})$ denotes the number of RNA–DNA interaction counts that RNA $r$ has with the bin $b$ at distance $\mathrm{ dist}$ to the gene locus of $r$. $\mathrm{ Count}(r, b)$ denotes the number of RNA–DNA interaction counts that RNA $r$ has with the bin $b$.

Since there are cases in which no distance can be computed (e.g. cross-chromosome interactions), a whole-genome bin-specific background is constructed in the same manner:


(2) \begin{eqnarray*}
\mathrm{ BG}_{\mathrm{ Bin}}(r, b) = \frac{\mathrm{ Count}(r, b)}{\sum _{r=1}^{m} \mathrm{ Count}(r, b)} \,\, ,
\end{eqnarray*}

where $m$ is the number of all bins on all chromosomes. Regions which are not covered by reads of an RNA $r$ are by definition set to $\mathrm{ Count}(r, b) = 0$.

The two background models are unified in a manner that favors the more conservative value for later testing:


(3) \begin{eqnarray*}
\mathrm{ BG}(r, b) = \max (BG_{\mathrm{ Bin}}(r, b), \mathrm{ BG}_{\mathrm{ Distance}}(r, \mathrm{ dist}(r, b))) \,\, ,
\end{eqnarray*}

where $\mathrm{ dist}(r, b)$ denotes the distance of the bin $b$ to the gene locus of $r$.

#### Identification of statistically robust RNA–DNA interactions

A particular RNA–DNA interaction $\mathrm{ count}(r,b)$ is assessed for statistical significance. Here an one-tailed Poisson test is used. The mean $\lambda$ is defined by incorporating the background count and the estimated RNA abundance value:


(4) \begin{eqnarray*}
\lambda = \mathrm{ BG}(r, b) * \mathrm{ Abund}(r) \,\, ,
\end{eqnarray*}

where $\mathrm{ Abund}(r)$ denotes the estimated RNA abundance in the dataset by summing over all RNA–DNA interactions of $r$:


(5) \begin{eqnarray*}
\mathrm{ Abund}(r) = \sum _{r=1}^{m} \mathrm{ Count}(r, b) \,\, .
\end{eqnarray*}

We conduct a Poisson test using significance level of $\alpha = 0.05$. To account for multiple testing, we apply the method described by Benjamini & Hochberg (1995)[29] and compute the corresponding FDR.

#### Snakemake pipeline assembly

*RADIAnT* is made publicly available as a Snakemake [30] pipeline. Input to the pipeline are split RNA and DNA FASTQ files, and a configuration file that can be modified by the user. The pipeline executes all necessary pre-processing steps (depletion of rRNA, processing of multimapping reads, assembly of complete RNA–DNA pairs, computation of RNA-bin interaction counts). If Red-C has been specified as the sequencing method, *RADIAnT* accounts for separate processing of the 3′ and 5′ parts of the RNA read in the aforementioned steps. The Snakemake pipeline subsequently applies the *RADIAnT* statistical method on the resulting RNA-bin counts. The outputs of the pipeline are RNA–DNA interactions called by *RADIAnT*, as well as several plots detailing important metrics and quality checks for the dataset.

### Assessing the consistency of *RADIAnT* interactions

#### Datasets

For the comparison of RNA–DNA interaction called by *RADIAnT* across different RNA–DNA ligation methods and experiments, RADICL-seq [19] and GRID-seq [21] data generated in mESCs were downloaded and analyzed. The NCBI GEO accession numbers for the RADICL-seq and GRID-seq data are GSE132192 and GSE82312, respectively, with there being two independent RADICL-seq experiments, one with 1% formaldehyde fixation and one with 2%.

#### Interaction calling with *RADIAnT*

Each of the independent replicates of each experiment was input to the *RADIAnT* Snakemake pipeline and analyzed with annotation data for the mm39 (GENCODE M35) genome assembly and 5 kb genomic bins. Interactions were then compared against one another locally by considering *Malat1*-DNA interactions in the genomic region surrounding the *Malat1* gene locus. Background interaction frequency, *Malat1* interaction frequency, and sites of significant (adjusted *P* < .05) *Malat1*-DNA interactions were plotted using ggplot2 per RNA–DNA ligation experiment.

#### Correlation between *RADIAnT* interactions per RNA–DNA ligation method

To test the consistency of interactions called by *RADIAnT* between RNA–DNA ligation methods, common interactions detected with at least 1 supporting read in each experimental replicate were determined. The adjusted *P* values calculated for these interactions by *RADIAnT* in each experimental replicate were then negative log-transformed, and the resulting values used as input to the R function *cor.test* in a pairwise manner. As a negative control, log-transformed adjusted *P* values were sampled in triplicate from the distribution of values calculated across the common interactions for all experimental replicates and assigned to the common interactions as a negative control. This process was carried out for *RADIAnT* interactions called against 5 and 25 kb bin sizes. Resulting pairwise Pearson correlation coefficients were plotted using ggplot2 (v3.5.0).

### Benchmarking of RNA–DNA interaction calling

#### Analysis of one-to-all RNA–DNA interaction data

Raw data corresponding to several one-to-all RNA–DNA interaction experiments were downloaded and analyzed accordingly. The datasets in question were *Malat1* RAP-DNA from mESCs [31] (GSE55914), along with *MALAT1* and *NEAT1* CHART data [32] from MCF-7 cells. For each dataset, reads were aligned to the appropriate genome build (mm39 GENCODE release M35 or hg38 GENCODE v47) using Bowtie2 [33] (v2.4.5) with the parameters –local and –very-sensitive. Coverage tracks were generated using bamCoverage [34] (v3.5.3) with the parameter –normalizeUsing CPM. Peaks were called using MACS [35] (v3.0.0), with the RNA–DNA alignments as treatment and the respective input samples as control.

#### RNA–DNA ligation data

For the comparison between RNA–DNA interaction calling methods, several RADICL-seq [19] and GRID-seq [21] datasets generated in mESCs and MDA-MB-231 cells were downloaded and analyzed with each RNA–DNA interaction caller. The NCBI GEO accession numbers for the datasets are GSE132192 (RADICL-seq mESCs) and GSE82312 (GRID-seq mESCs and MDA-MB-231), respectively.

#### Analysis with *RADIAnT*

Both RADICL-seq and GRID-seq reads were used as input to the *RADIAnT* Snakemake pipeline in order to call interactions. Depending on the input data, either mm39 (GENCODE M35 release) or hg38 (GENCODE v47) genome assemblies were used in combination with 25, 50, or 100 kb genomic bins. RNA-bin interaction counts were pooled across replicates prior to interaction calling with *RADIAnT*. Statistically significant interactions were designated as those with an adjusted *P* < .05.

#### Implementation of Bonetti *et al*. statistical method

The computational method for calling RNA–DNA interactions as proposed by Bonetti *et al*. [19] was reimplemented in R. Briefly, RNA-bin counts from the identical pre-processing to the *RADIAnT* analysis were subjected to a binomial test where the parameters were as follows: $x$ was the RNA-bin interaction count, $n$ was the sum of all RNA-bin interaction counts for the RNA in question, $p$ is the reciprocal of the total number of bins detected as being bound by the RNA, and $\mathrm{ alternative}$ was set to “greater.” Resulting *P* values were adjusted for multiple testing using the Benjamini–Hochberg procedure [29].

#### Implementation of Zhou *et al*. statistical method

The computational method for calling RNA–DNA interactions as proposed by Zhou *et al*. [36] was also reimplemented in R. Briefly, RNA-bin counts as detected by RADIAnT pre-processing were subset to *trans*-chromosomal interactions of mRNAs only. A bin-specific background is constructed from the number of reads at each 1 kb bin, which is normalized by the overall number of genes in the interaction set. The normalized read count is then smoothed in a moving window approach (10 bins), further normalizing by total read count per chromosome and number of bins per chromosome, yielding a bin-specific background coverage. The approach by Zhou *et al*. calculates the fold enrichment as the ratio of the observed RNA-bin interaction frequency (normalized by read count per chromosome and number of bins per chromosome) to the background coverage.

#### Calling of RNA–DNA interactions using *BaRDIC*

*BaRDIC* was installed and run as per the instructions at https://github.com/dmitrymyl/BaRDIC/. Genomic annotation and chromosome sizes were supplied based on mm39 (GENCODE M35) or hg38 (GENCODE v47) as appropriate for the respective dataset, and the background RNAs used were mRNAs as classified by GENCODE, which were present in the dataset.

#### Performance evaluation

Each RNA–DNA interaction calling method was implemented as described above. RADICL-seq and GRID-seq data were subjected to identical pre-processing procedures prior to interactions being called by each method.

Classification performance evaluation of each of the tools was carried out in R using the *ROCR* (v1.0.11) package. The evaluation dataset was composed of genomic bins which either intersected with a one-to-all RNA–DNA peak or not. For each method, a predictor value was assigned per bin. These values were negative log-transformed adjusted *P* values for *RADIAnT, BaRDIC*, and Bonetti *et al*., and fold enrichment for Zhou *et al*. The relative performance of each tool was then computed with *ROCR*, comprising of receiver operating characteristic (ROC) analysis and precision-recall analysis.

Correlative performance of each RNA–DNA interaction caller was conducted using an adapted form of gene set enrichment analysis (GSEA). Briefly, genomic bins were ranked according to the RNA–DNA interaction statistic output of each caller. Hits were considered as bins intersecting with at least one one-to-all RNA–DNA interaction peak. The R package *fgsea* [37] was then used to conduct the GSEA, resulting in an enrichment score per method, per dataset.

#### Memory and time profiling

Memory and runtime performance were evaluated on a Linux server running Ubuntu 22.04.5 LTS with dual AMD EPYC 745 32-core processors, providing a total of 64 physical cores and 128 logical threads. The system was equipped with 503 GB of RAM and 99 GB of swap space. At the time of profiling, the available memory was ~495 GB.

For the sake of comparability, we aimed to conduct memory and runtime performance of *RADIAnT* and *BaRDIC* on the same input set. *BaRDIC* by default, discards any genes with <1000 interaction counts (mcon parameter set to 1000). *RADIAnT*, by default, applies a minimum interaction count threshold of 2. In initial tests, reducing *BaRDIC’*s *mcon* parameter to 2 (as well as 10, 25, and 50) led to excessive memory consumption and frequent crashes due to resource limits. As *BaRDIC* executed reliably only at mcon=100, this threshold was used for all performance comparisons. Accordingly, *RADIAnT* was provided with a matched count input set filtered to include the same genes meeting the 100-count threshold. For the replicate n1 of the RADICL-seq of mESCs at 2% formaldehyde, this resulted in a set of 11 961 genes involved in 829 493 interactions supported by 23 071 070 reads in total. *BaRDIC* was run on 16 cores, while the interaction calling element of *RADIAnT* is predominantly uses a single core (although the R package *data.table* can take advantage of multicore processing).

### Cell culture

Human umbilical vein endothelial cells (HUVEC, batches 405Z013, 408Z014, 416Z042, Lonza) were cultured on 0.2% gelatin-coated dishes in endothelial growth medium (EGM, Pelo Biotech). EGM was supplemented with 8% fetal calf serum (FCS, Sigma–Aldrich), 50 U ml^−1^ penicillin (Gibco), and 50 µg ml^−1^ streptomycin (Gibco). The cells were thawed freshly and passaged once on a 10 cm dish (Sarstedt). Experiments were performed with passage 3. Cells were incubated at 37°C with 5% CO_2_. If not stated otherwise, three batches of HUVECs were used for the experiments. All media used were pre-warmed at 37°C.

### Endothelial-to-mesenchymal transition

EndMT was induced according to [38]. HUVECs were seeded in EGM (Pelo Biotech) with a density of 6900 cells cm^−2^ on 0.2% gelatin-coated dishes (15 cm, Sarstedt) 1 day prior to induction of EndMT. To induce EndMT, differentiation medium (DM) was used, which consisted of endothelial basal medium (Pelo Biotech) supplemented with 8% FCS (Sigma–Aldrich), 50 U ml^−1^ penicillin (Gibco), 50 µg ml^−1^ streptomycin (Gibco), and L-glutamine (Gibco). 10 ng ml^−1^ TGF-$\beta$ 2 (Peprotech) and 1 ng ml^−1^ IL-1$\beta$  (Peprotech) were added freshly to pre-warmed DM medium. Medium was changed every 24 h for a total of 5 days.

### Nuclear RNA sequencing

Nuclear RNA sequencing was performed on control HUVECs and HUVECs stimulated to undergo EndMT. To isolate nuclear extract, medium was aspirated, and cells were washed once with cold Hank’s solution. Harvesting was performed by scraping cells twice in 500 µl Hank’s solution and centrifuging (1 min, 13 000 rpm at 4°C). The supernatant was removed, and the pellet resuspended in 600 µl buffer A (10 mM HEPES, pH 7.9, 10 mM KCl, 0.1 mM ethylenediaminetetraacetic acid (EDTA), 0.2 mM ethylene glycol tetraacetic acid (EGTA), 1 mM dithiothreitol (DTT), 40 µg ml^−1^ phenylmethylsulfonyl fluoride (PMSF), 12 µg ml^−1^ protease inhibitor mix). After incubation on ice for 15 min, 45 µl 10% Nonidet (Sigma–Aldrich) were added to lyse the cytosolic membrane, the cells were vortexed for 10 s, and centrifuged (1 min, 13 000 rpm at 4°C). The supernatant, containing the cytosolic fraction, was removed, and the pellet resuspended in 150 µl buffer C (20 mM HEPES, pH 7.9, 0.4 M NaCl, 1 mM EDTA, 1 mM EGTA, 1 mM DTT, 40 µg ml^−1^ PMSF, 12 µl ml^−1^ protease inhibitor mix). To lyse the nuclei, the resuspended pellet was rocked for 15 min at 1400 rpm and 4°C. To remove insoluble debris, the lysate was centrifuged (5 min, 13 000 rpm at 4°C), and the supernatant was transferred to a new 1.5 ml tube.

RNA isolation was performed according to Bio&SELL RNA isolation kit manual. If not otherwise stated, RNA was eluted in 30 µl at 13 000 rpm for 1 min at room temperature. RNA concentration was measured with the NanoDrop ND-1000. Library preparation was carried out using the SMARTer^®^ Stranded Total RNA-seq Kit v2–Pico input mammalian. Libraries were sequenced on an Illumina NextSeq 500 sequencer with single-end, 75 bp read length and to an average depth of 30 million reads.

### Nuclear RNA sequencing analysis

Nuclear RNA sequencing reads were aligned to the hg38 (GENCODE v43) genome assembly using STAR [25] (v2.7.10) with the parameters –outSAMtype BAM SortedByCoordinate –quantMode GeneCounts. Differential gene expression analysis was performed using DESeq2 (v1.40.2) [39], with the respective donor per sample included in the experimental design.

### Assay for transposase accessibility-sequencing

Assay for transposase accessibility (ATAC) resuspension buffer was prepared (10 mM Tris–HCl, pH 7.4, 10 mM NaCl, 3 mM MgCl_2_). Fifty thousand cells per biological replicate for control and EndMT conditions were washed once with 50 µl ice-cold phosphate buffered saline (PBS) and lysed in 50 µl cold ATAC lysis buffer (97% ATAC resuspension buffer, 0.1% NP-40, 0.1% Tween20, 0.01% Digitonin). Cells were incubated for 3 min on ice before being topped up to 1 ml with ATAC wash buffer (99% ATAC resuspension buffer, 0.1% Tween20) and inverted three times. Nuclei were pelleted by centrifugation (10 min, 500 × *g*, 4°C). Supernatants were removed, and nuclei were resuspended in 50 µl ATAC transposition reaction mix (25 µl 2× Tagment DNA buffer (Illumina), 2.5 µl Transposase (Illumina), 22.5 µl nuclease-free water). After incubation (37°C, 1000 rpm, 30 min), the DNA was purified using the Qiagen MinElute kit and eluted in 10 µl elution buffer. The library was prepared with the ATAC library amplification reaction mix, as described in [40]. Following amplification, the library was sequenced on an Illumina NextSeq 500 sequencer with paired-end, 75 bp read length, and to an average depth of 15 million reads.

Resulting sequencing reads were aligned to the hg38 genome assembly (GENCODE v43) using Bowtie2 [33] (v2.4.5) with the parameters –local and –very-sensitive. Coverage tracks were generated using bamCoverage [34] (v3.5.3) with the parameter –normalizeUsing CPM. Dynamic ATAC-seq peaks between control and EndMT conditions were detected using MACS [35] (v3.0.0) bdgdiff, with filtered sequencing depths per condition used to normalize between samples.

### RADICL-seq

RADICL-seq was performed as reported in [19] on control HUVECs and HUVECs subjected to EndMT. For each condition, 4 million cells were washed once with PBS, trypsinated, and spun down (1200 rpm, 4 min). The cell pellet was resuspended in 5 ml PBS, and the cells were counted using a Neubauer counting chamber and Trypan blue staining. Two million cells were cross-linked through resuspension in 2 ml fresh 1% formaldehyde for 10 min at room temperature. Crosslinking was stopped by adding 715 µl of freshly prepared 1 mol l^−1^ glycine in double-distilled water. Cells were centrifuged (100 × *g*, 5 min, 4°C), and the cell pellet was washed once with 5 ml ice-cold PBS and spun down again (100 × *g*, 5 min, 4°C). Pellets were shock-frozen in liquid nitrogen and stored at −80°C. Thereafter, the RADICL-seq protocol was carried out as described by Bonetti *et al*. [19]. Final library concentrations were measured by Qubit fluorometer, and library quality and size was determined by a Bioanalyzer High Sensitivity DNA Analysis according to manufacturer’s protocol (Agilent). Paired-end sequencing with 150 bp read length was carried out using an Illumina NextSeq 500 sequencer with a sequencing depth of 125 million reads per condition.

### Analysis of EndMT RADICL-seq data

Control and EndMT RADICL-seq data were analyzed using the *RADIAnT* Snakemake pipeline, using the hg38 genome assembly and 25 kb genomic bins. Significant RNA–DNA interactions were those with a *RADIAnT P* value < .05. RNA biotypes were assigned to populations of interacting RNAs as annotated in GENCODE release 43. Interaction proportions per RNA were calculated per condition by taking the number of significant interactions per RNA divided by the total number of significant interactions in the condition. Circular genome plots were made using ggplot2 (v3.5.0).

### siRNA-mediated knockdown of lncRNAs

For the knockdown of lncRNAs with small interfering RNA (siRNA), HUVEC were seeded at 15 000 cells/cm^2^ 1 day before transfection. Cells were transfected using Lipofectamine RNAiMAX according to the instructions provided by Thermo Fisher Scientific. A custom Silencer™ Select siRNA library (Thermo Fisher Scientific, Cat. No. 4392426) was used for individual knockdowns of lncRNA targets (*GAS5, NUTM2B-AS1*, and *MIR222HG*), with transfection performed using 25 nM siRNA. Silencer™ Select Negative Control No. 1 siRNA (Thermo Fisher Scientific, Cat. No. 4390844) served as a control. Cell medium was changed to EGM after 4 h and again the next day. All siRNA experiments were performed for 48 h.

### Endothelial proliferation assay

Proliferation of HUVECs treated with either control or lncRNA-targeting siRNAs was monitored for 96 h using the IncuCyte S3 live cell imaging system (Sartorius). To do this, HUVECs were plated at a density of 4000 cells/well in a 96-well plate in endothelial growth medium (Gibco). The cells were then transfected with appropriate siRNA in endothelial basal medium (Gibco) as described earlier. The following day, media was changed to endothelial growth medium (Gibco) prior to the proliferation assay. Proliferation was measured by the detection of Nuclight Rapid Red dye (Sartorius), which was added to the cells 20 min prior to the beginning of the IncuCyte run. The plate was scanned every 3 h for 120 h, and the fluorescence quantified. Changes in the proliferation rate were calculated by calculated the time at which the cells reached 50% of their maximum confluency per well, and then comparing these values between knockdown and control conditions.

### Total RNA-sequencing

Nine hundred nanograms of total RNA isolated from HUVECs was used as input for SMARTer Stranded Total RNA Sample Prep Kit—HI Mammalian (Takara Bio). Sequencing was performed on the NextSeq 500 instrument (Illumina) using v2 chemistry, resulting in an average of 34 million reads per library with 2 × 75 bp paired-end setup. The resulting raw reads were assessed for quality, adapter content, and duplication rates with *FastQC* [41]. *Trimmomatic* version 0.39 was employed to trim reads after a quality drop below a mean of Q20 in a window of 10 nucleotides [42]. Only reads between 30 and 150 nucleotides were cleared for further analyzes. Trimmed and filtered reads were aligned versus the Ensembl human genome version hg38 (release 99) using *STAR* 2.7.3a with the parameter “–outFilterMismatchNoverLmax 0.1” to increase the maximum ratio of mismatches to mapped length to 10 [25]. The number of reads aligning to genes was counted with *featureCounts* 1.6.5 tool from the Subread package [43]. Only reads mapping at least partially inside exons were admitted and aggregated per gene. Reads overlapping multiple genes or aligning to multiple regions were excluded. Differentially expressed genes (DEGs) were identified using *DESeq2* version 1.26.0 [39]. Only genes with a minimum fold change of ±1.5 (log_2_ ± 0.59), a maximum Benjamini–Hochberg corrected *P*-value of .05, and a minimum combined mean of five reads were deemed to be significantly differentially expressed. The Ensembl annotation was enriched with UniProt data (release 06.06.2014) based on Ensembl gene identifiers (Activities at the Universal Protein Resource (UniProt) [44]).

### Combined analysis of RADICL-seq, RNA-seq, and scRNA-seq

The combined analysis was conducted on lncRNA-regulated genes, which were defined as genes where the gene locus overlapped with a lncRNA-bound locus, as called by *RADIAnT* on EndMT RADICL-seq data, and which were differentially expressed in RNA-sequencing data (adjusted *P* < .05) after siRNA-mediated knockdown of the respective lncRNA. Statistical enrichment of lncRNA-bound genes in the DEG population, compared to the non-differential population, was computed using a Chi-squared test.

Term enrichment analysis on lncRNA-regulated genes was performed using Metascape [45] (https://metascape.org). A list of putative RNA-regulated genes per candidate RNA (*GAS5, MIR222HG*, and *NUTM2B-AS1*) was generated as Metascape input. Metascape queried multiple ontology sources, including Gene Ontology Biological Processes, Reactome, KEGG, WikiPathways, and PANTHER. The complete gene set served as background for the enrichment analysis. A term was reported as enriched at *P* < .01, a minimum count of 3, and an enrichment factor >1.5.

A gene module was constructed per putative set of RNA up- and downregulated genes. Relative gene module expression was queried in a single-cell RNA-sequencing experiment where HUVECs were induced to undergo EndMT [46] (GEO accession number *GSE143151*) using *Seurat* [47]. Epigenetic characteristics of the lncRNA-bound gene loci were examined in more detailed using a ChromHMM model [48] learned from epigenetic data obtained from HUVECs [14].

## Results

### RNA–DNA ligation data are confounded by distance and abundance

**Figure 1.**
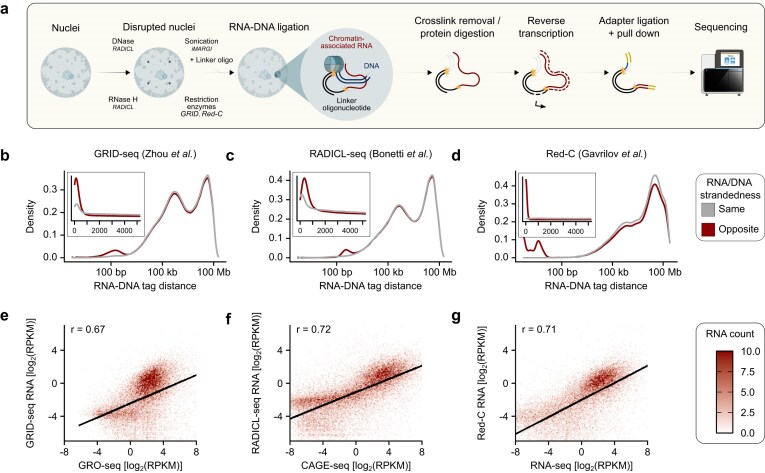
RNA–DNA ligation data are confounded by distance and RNA abundance. (**a**) Generalized workflow for RNA–DNA ligation experiments, which comprise of introduction of a tagged linker oligonucleotide to disrupted nuclei, which bridges RNA and DNA in proximity, resulting in chimeric RNA–DNA sequencing reads. (**b**–**d**) RNA–DNA tag distances in base pairs from published GRID-seq [36], RADICL-seq [19], and Red-C [20] experiments, split by strandedness. (**e**–**g**) Pearson correlation between RNA abundance in RNA–DNA ligation data and RNA expression as captured by GRO-seq [21], CAGE [19], or RNA-seq [20].

Published RADICL-seq [19], GRID-seq [21], and Red-C [20] datasets were downloaded, split into paired RNA and DNA sequences, and aligned to appropriate genome assemblies. For each RNA–DNA read pair that mapped to the same chromosome, the linear distance between the mapped RNA and DNA was calculated in base pairs. Upon plotting the density of RNA–DNA distances by strandedness, a clear enrichment could be observed in RNA–DNA distances <1 kilobase where strandedness was opposite (Fig. 1b–d). This corresponds to a high proportion of RNA associating with complementary DNA in its own transcriptional locus, which is likely a result of nascent transcription.

Given the potential impact of active transcription on the data, the relationship between RNA expression and abundance in RNA–DNA ligation data was also investigated. For each ligation method, accompanying gene expression data were also downloaded and analyzed. In each case, significant positive correlations between RNA expression and abundance in the ligation data could be observed (Fig. 1e–g).

These two observations made it clear that both the abundance of the RNA and the RNA gene locus-binding site distance should be considered when analyzing these data. Additionally, to better control for variation between methods and potential quality differences between experiments, the statistical background should be built from the given dataset, rather than in a generalized manner.

### *RADIAnT* calls RNA–DNA interactions against a unified, dataset-specific background

Based on the observations described earlier, we formulated *RADIAnT. RADIAnT* uses the RNA–DNA ligation data provided by the user to build a unified, dataset-specific statistical background, against which RNA–DNA interactions are called. To aggregate interaction counts and enable quantification of the data, the genome is split into bins of a user-defined size (e.g. 5 kb). RNA–DNA interaction counts are then summarized as the total observed reads mapping to an RNA-bin combination. By dividing the RNA-bin interaction count by the total counts for the RNA in question, the interaction frequency of the RNA at the given bin can be derived, a metric that is robust to expression differences between RNAs.

**Figure 2.**
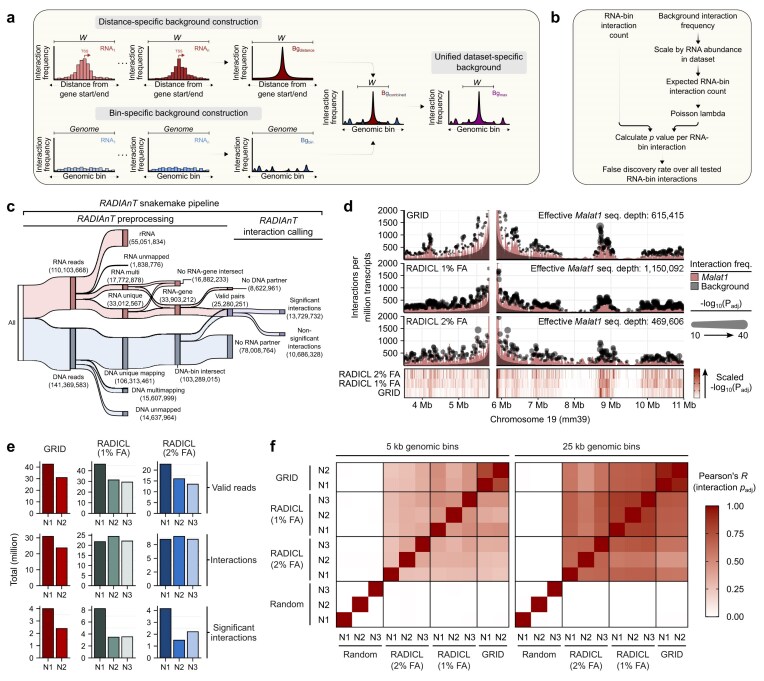
*RADIAnT* calls consistent RNA–DNA interactions regardless of input data type. (**a, b**) *RADIAnT* utilized the input data to build distance- and bin-specific statistical backgrounds, which are merged into a single unified background against which RNA–DNA interactions can be called in an RNA-specific manner. (**c**) *RADIAnT* interaction calling is incorporated into a reads-to-interactions Snakemake pipeline for complete processing of diverse RNA–DNA ligation data types. The example output Sankey plot is from a GRID-seq experiment conducted in mESCs [21]. (**d**) Local *Malat1*-DNA interactions called using *RADIAnT* from GRID-seq [21], RADICL-seq (1% formaldehyde), and RADICL-seq (2% formaldehyde) [19] data generated from mESCs. (**e**) Total numbers of valid paired RNA–DNA reads, unique RNA–DNA interactions, and statistically significant (adjusted *P* < .05) RNA–DNA interactions called by *RADIAnT*, for each replicate of the GRID-seq and RADICL-seq experiments from mESCs (denoted N1–3). (**f**) Pearson correlation coefficients between adjusted *P* values calculated by *RADIAnT* for commonly observed interactions between replicates (denoted N1–3) of GRID-seq and RADICL-seq experiments in mESCs, as well as randomly sampled adjusted *P* values, for 5  and 25 kb genomic bin sizes.

To account for the effect of nascent transcription, a distance-specific background is constructed from the input dataset. This is done by calculating the mean RNA-bin interaction frequency at defined linear distances from the RNA gene locus (Fig. 2a, top). The mean of these frequencies per distance across all RNAs in the dataset is then taken as the distance-specific *RADIAnT* background for the experiment in question.

In a separate approach designed to control for the overrepresentation of specific genomic bins resulting from technical artifacts or sequence biases, *RADIAnT* also constructs a bin-specific background. This is done by calculating the mean RNA interaction frequency at each genomic bin (Fig. 2a, bottom). Only interaction frequencies where the RNA in question originates from a different chromosome—and are therefore not subject to linear distance bias—are considered when building bin-specific background.

The two backgrounds are then combined (Fig. 2a, right). Consequently, for every genomic bin and a given RNA, there exists a bin-specific background RNA interaction frequency in addition to a distance-based background interaction frequency (imposed if the RNA in question originates from the same chromosome as the bin). These two backgrounds are unified by taking the maximum expected interaction frequency per bin of the genome, ensuring maximum stringency. The output of these steps is an expected interaction frequency of each RNA in the dataset at each genomic bin.

To call interactions for a given RNA (Fig. 2b), the unified background interaction frequency is scaled by the matching RNA abundance in the input data, giving an expected RNA-bin interaction count. The observed RNA-bin count is compared against the expected RNA-bin count in a Poisson test, resulting in a *P* value for the RNA-bin interaction. The interaction *P* value is adjusted for multiple testing according to the total tested RNA-bin combinations. By default, this encompasses any RNA-bin interaction with at least two supporting reads. Interactions with an adjusted *P* value < .05 are taken as significant.

*RADIAnT* is incorporated into a reads-to-interactions Snakemake [30] pipeline (Fig. 2c), which takes split sequencing reads generated from RNA–DNA ligation experiments as input. The pipeline conducts appropriate pre-processing and returns called RNA–DNA interactions as described earlier. By implementing the pipeline on different RNA–DNA ligation experiments from the same cell type, the capability of *RADIAnT* to adapt to different input data while maintaining consistent interaction calling was assessed.

### *RADIAnT* calls consistent RNA–DNA interactions regardless of input data type

To determine the consistency of *RADIAnT* interaction calling, independent RNA–DNA ligation experiments from the same biological context were utilized. This entailed two triplicate RADICL-seq experiments (1% and 2% formaldehyde fixation) [19] and a duplicate GRID-seq experiment [21], all conducted in mESCs.

For an assessment of how consistently *RADIAnT* calls interactions against a predominantly distance-specific background, several megabases surrounding the gene locus of *Malat1* were considered. *Malat1* is an extensively studied nuclear-localized lncRNA; it is highly conserved, ubiquitously expressed, and has important regulatory functions in transcription and alternative splicing [49]. In spite of the different methods, experimental conditions, and sequencing depths, similar patterns of *Malat1*-DNA interactions were called by *RADIAnT* across the datasets (Fig. 2d). Most notably, highly significant interactions were called across a region between 8.75 and 9 Mb in all three datasets, with interactions in this region being called as more significant than interactions closer to the gene locus, indicating the functionality of the distance-specific *RADIAnT* background.

For a genome-wide perspective, the numbers of valid paired RNA–DNA reads, RNA-bin interactions, and statistically significant RNA–DNA interactions called by *RADIAnT* were compared across the experiments. An expected relationship between the number of valid reads and number of significant interactions could be observed (Fig. 2e). However, this was not due to increased numbers of interactions in general.

To test genome-wide concordance between called interactions, negative log-transformed adjusted *P* values for common interactions detected (read count > 0) across all the experimental replicates were compared. Comparisons were made between RNA-bin interactions at higher (5 kb) and lower (25 kb) resolutions. In each case, the interactions called by *RADIAnT* were strongly correlated across the independent experiments and replicates, except for randomly sampled values, which acted as a negative control (Fig. 2f). Whilst those interactions called using 25 kb bins were more concordant, those called with 5 kb bins were still consistent across the experiments. Given the likely relationship between sequencing depth and maximal resolution, genomic bin size is a user-defined parameter in the *RADIAnT* pipeline, which can be tuned based on the depth and quality of the data in question.

While *RADIAnT* could call consistent RNA–DNA interactions across different experimental contexts, the question remained whether the interactions called by *RADIAnT* were biologically meaningful, and how well they reflected interactions identified by orthogonal one-to-all RNA–DNA interaction methods. To address this, interactions called by *RADIAnT* were compared against those detected in one-to-all data for several combinations of RNAs, datasets and bin sizes. Additionally, *RADIAnT* interactions were compared to those called by alternative methods proposed for analysis of all-to-all RNA–DNA ligation data.

### *RADIAnT* outperforms other all-to-all RNA–DNA interaction callers

**Figure 3.**
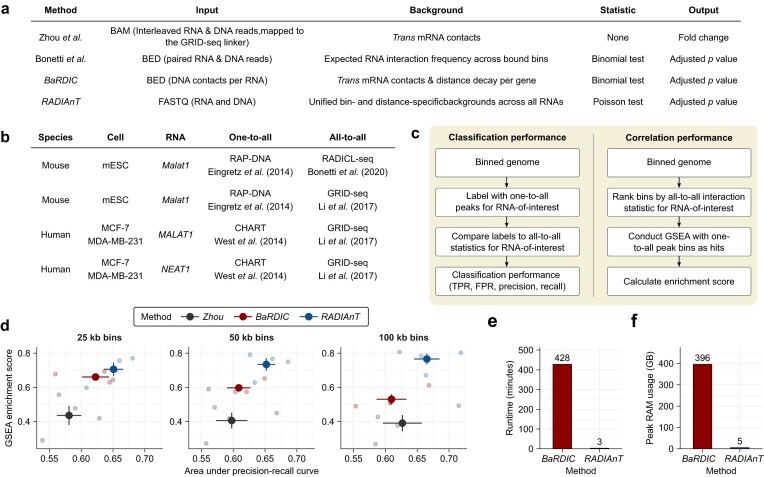
*RADIAnT* outperforms other RNA–DNA interaction callers in detection of one-to-all RNA binding sites. (**a**) RNA–DNA interaction calling methods whose performance was evaluated. (**b**) One-to-all and all-to-all dataset combinations used to test the relative performance of the different RNA–DNA interaction callers. (**c**) Workflows for evaluating the classification and correlative performance of each RNA–DNA interaction caller, using the respective matched one-to-all and all-to-all RNA–DNA binding data. (**d**) GSEA enrichment score and area under the precision-recall curve for each RNA–DNA interaction caller as evaluated on each all-to-all and one-to-all dataset combination across 25, 50, and 100 kb genomic bins. Large points denote mean values, with whiskers denoting the standard error of the mean. Individual points represent the values for each dataset combination tested. (**e**) Runtime in minutes of *BaRDIC* and *RADIAnT* interaction calling on a single RADICL-seq sample. (**f**) Peak random access memory (RAM) usage during *BaRDIC* and *RADIAnT* interaction calling on a single RADICL-seq sample.

To assess the ability of *RADIAnT* to identify specific RNA–DNA interactions, one-to-all RNA–DNA interaction data were used to evaluate the relative performance in the accurate detection of ground truth RNA–DNA interactions of the method compared to other RNA–DNA interaction callers. The callers used in this comparison were *RADIAnT, BaRDIC* [24], the method proposed by Zhou *et al*. for analysis of GRID-seq data [21], and the method proposed for analysis of RADICL-seq by Bonetti *et al*. (Fig. 3a). Several datasets were used in the evaluation. Namely, one-to-all data included were *Malat1* RAP-DNA generated from mESCs [31] were compared to *Malat1*-DNA interactions called from either RADICL-seq or GRID-seq data in the same cell type, and one-to-all CHART data for *NEAT1* and *MALAT1* in MCF-7 cells was used as ground truth for testing the performance of interaction calling in MDA-MB-231 cells (Fig. 3b).

The performance of the RNA–DNA interaction callers was tested in two ways. In the first, the relative classification performance of each method was evaluated by using the one-to-all data to label bins of the genome, which were compared to the output statistic of each method in order to derive true positives, true negatives, false positives, and false negatives. The rate of these at alternative thresholds permits receiver operating characteristic and precision-recall analyses (Fig. 3c, left). In the second way, the correlative performance of each method was tested using an adapted form of GSEA [50] (Fig. 3c, right). Briefly, genomic bins were ranked by the output statistic of each tool, with bins containing a one-to-all peak taken as hits. GSEA was then used to determine the relative enrichment of hits at the top of the ranked list, described by an enrichment score.

In each of the approaches described earlier, across each combination of one-to-all and all-to-all datasets and differing bin sizes (25, 50, and 100 kb), *RADIAnT* outperformed each of the other RNA–DNA interaction callers (Fig. 3d, Supplementary Fig. S1). *BaRDIC* consistently performed second best of the tested callers, followed by the method from Zhou *et al*., and finally that from Bonetti *et al.. RADIAnT* interaction calling performance also increased with increasing bin size, suggesting that the method scales with relative sequencing depth.

The final performance measure evaluated here was the relative computational requirements of *RADIAnT* and *BaRDIC*, which were the two highest-performing methods in classification and correlation of RNA-binding sites with one-to-all data. On a test RADICL-seq sample [19], *BaRDIC* required 428 min and 396 GB of memory to call RNA–DNA interactions for all RNAs with at least 1000 reads in the dataset, compared to 3 min and 5 GB of memory required by *RADIAnT* (Fig. 3e-f).

Taken together, these evaluation experiments demonstrated that *RADIAnT* was the most accurate and most efficient RNA–DNA interaction caller available at the time of writing. However, the ability of *RADIAnT* to detect dynamic, biologically meaningful RNA–DNA interactions in a physiological context remained unexplored.

### Detection of dynamic RNA–DNA interactions during EndMT using *RADIAnT*

**Figure 4.**
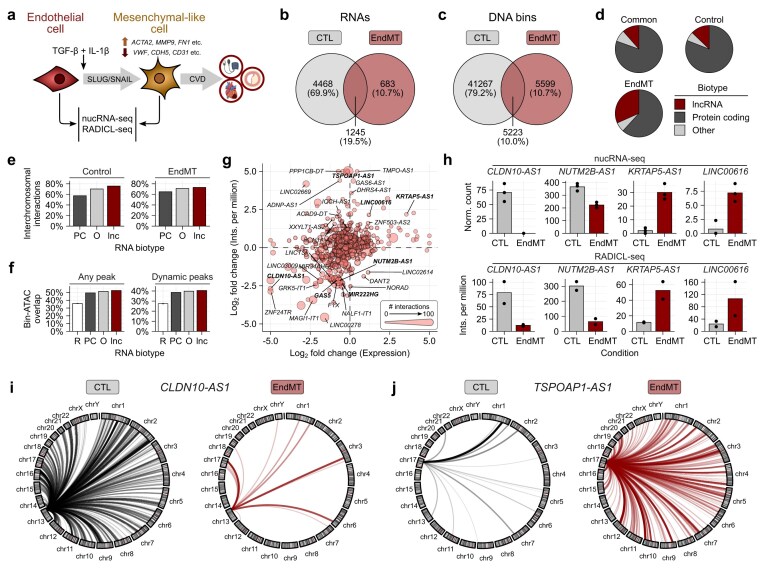
Detection of dynamic RNA–DNA interactions during EndMT using RADIAnT. (**a**) Overview of EndMT reproduced experimentally by TGF-$\beta$2 and IL-1$\beta$ treatment of HUVECs. (**b, c**) Unique and common interacting RNAs and interacting DNA bins in control and EndMT conditions. (**d**) RNA biotype proportions in control, common, and EndMT RNA populations. (**e**) Percentage of interchromosomal RNA–DNA interactions for protein-coding RNAs (PC), lncRNAs, and other RNA biotypes (O). (**f**) Percentage of random (R), protein-coding (PC), other (O), or long non-coding (lnc) RNA biotype-bound DNA bins overlapping with any ATAC-seq peaks detected in control and EndMT conditions, or dynamic ATAC-seq peaks between the conditions. (**g**) LncRNAs stratified by change in expression measured by nucRNA-seq and change in DNA interactions (interactions per million) as detected by RADICL-seq between control and EndMT conditions. LncRNAs further analyzed in subsequent figures are labeled in bold. (**h**) Expression changes and RNA–DNA interaction proportion changes between control and EndMT conditions for the lncRNAs *CLDN10-AS1, NUTM2B-AS1, KRTAP5-AS1*, and *LINC00616*. (**i**) RNA–DNA interactions called for *CLDN10-AS1* in control and EndMT conditions. (**j**) RNA–DNA interactions called for *TSPOAP1-AS1* between control and EndMT conditions.

To test the ability of *RADIAnT* to identify dynamic RNA–DNA interactions between biological settings, EndMT was used as a model. In EndMT, endothelial cells transdifferentiate to a more proliferative and invasive mesenchymal-like phenotype, which has been linked to physiological and pathological processes in the cardiovascular system [51, 52]. In an *in vitro* model of EndMT, HUVECs were treated with interleukin-1 beta (IL-1$\beta$) and transforming growth factor beta 2 (TGF-$\beta$2) to induce a phenotypic switch (Fig. 4a). Nuclear RNA-sequencing (nucRNA-seq), RADICL-seq, and ATAC-seq were performed on control (endothelial) and treated (EndMT) cells.

When comparing RNA and DNA populations involved in *RADIAnT* RNA–DNA interactions detected in two biological replicates of control or EndMT conditions, condition-specific and common features could be extracted (Fig. 4b and c). When examining these populations, there was a greater proportion of lncRNAs involved in interactions in EndMT compared to common or control conditions (Fig. 4d). LncRNAs were also involved in greater proportions of interchromosomal RNA–DNA interactions across control and EndMT condition (Fig. 4e), as well as interacting at a greater proportion of regions where ATAC-seq peaks were detected compared to random bins, protein-coding RNA-bound bins or other RNA biotype-bound bins (Fig. 4f).

To identify dynamic chromatin-associated lncRNAs in EndMT, nucRNA-seq data were integrated with the RNA–DNA interactions called by *RADIAnT*, with the proportion of the total interactions per condition being used as a metric to measure RNA–DNA binding changes. A number of lncRNAs whose expression and interaction proportion were dynamic between the conditions were detected (Fig. 4g), among them *CLDN10-AS1, NUTM2B-AS1, KRTAP5-AS1*, and *LINC00616* (Fig. 4h). Some lncRNAs demonstrated highly dynamic changes in chromatin association between control and EndMT conditions. Notably, this was the case for lncRNAs that show an almost complete loss in DNA binding in EndMT (e.g. *CLDN10-AS1*), as well as for lncRNAs that display increased chromatin interaction after EndMT (e.g. *TSPOAP1-AS1*) (Fig. 4i and j).

This use case demonstrates that *RADIAnT* can be utilized to examine dynamic biological processes and identify putative RNA regulators involved in physiological and pathological processes. To decompose the complex RNA–DNA binding behaviors seen here and identify putative RNA-regulated gene modules, individual lncRNAs were further interrogated for their potential gene regulatory functions.

### *RADIAnT*-called interactions underlie putative RNA-dependent regulatory networks

To further investigate the functional roles of RNA–DNA interactions called using *RADIAnT* during the course of EndMT, candidate chromatin-associated lncRNAs were selected for further mechanistic investigation. To do this, dynamically chromatin-associated lncRNAs were stratified by their changes in RADICL-seq interactions and nucRNA-seq expression, as well as the effects on endothelial proliferation following siRNA-mediated knockdown of the lncRNA. Based on these metrics, *GAS5, MIR222HG*, and *NUTM2B-AS1* were selected for further study, each of which displayed downregulation of expression and chromatin association during EndMT and had consistent effects on endothelial proliferation following their knockdown (Supplementary Fig. S2a).

**Figure 5.**
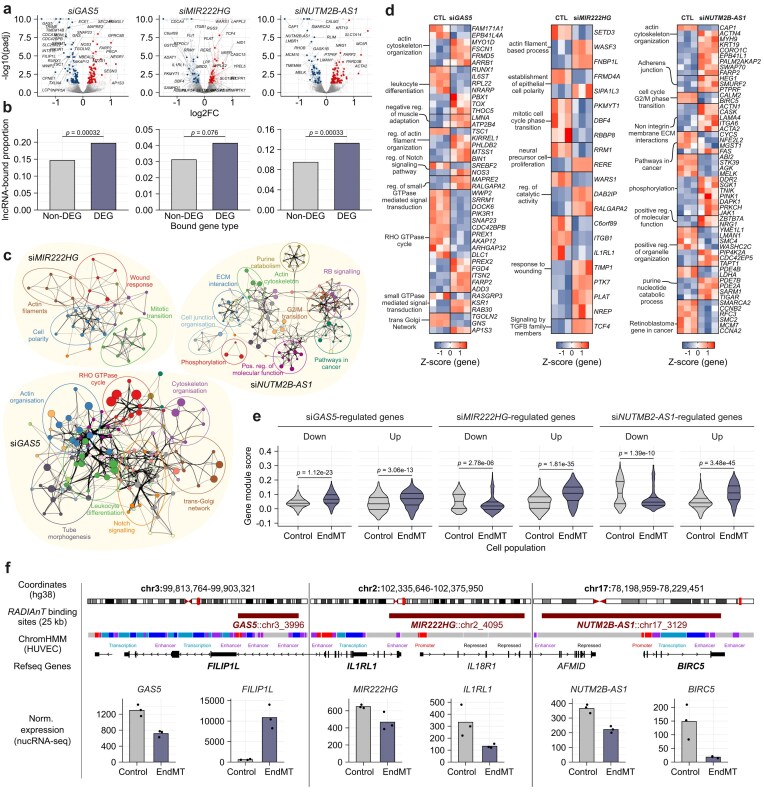
Functional validation of lncRNAs identified by *RADIAnT* to show dynamic chromatin interactions during EndMT. (**a**) Differential gene expression analysis following siRNA-mediated knockdown of *GAS5, MIR222HG*, and *NUTM2B-AS1* in HUVECs. Highlighted points indicate genes whose loci intersect with a respective RNA–DNA binding site as called by *RADIAnT* from RADICL-seq data. (**b**) Statistical enrichment of RNA-bound loci in the DEG population of each lncRNA knockdown RNA-seq experiment compared to the respective non-DEG population, computed by a Chi-squared test. (**c**) Metascape analysis illustrating enriched sets of terms computed from target genes of each lncRNA. (**d**) Z-score scaled expression of lncRNA gene targets in CTL and siRNA-mediated knockdown of *GAS5, MIR222HG*, or *NUTM2B-AS1*, grouped by enriched pathways/processes as computed by Metascape. (**e**) RNA target gene module expression in control or EndMT-induced cells in a scRNA-seq experiment [46], per candidate lncRNA and per up- or downregulation following siRNA-mediated depletion of the respective lncRNA. Module expression differences were tested with an unpaired, two-sided Wilcoxon test with Benjamini–Hochberg adjustment. (**f**) Selected target gene loci for *GAS5, MIR222HG*, and *NUTM2B-AS1* showing *RADIAnT*-called lncRNA binding sites, chromatin state annotations as per a ChromHMM model learned from epigenomic data generated from HUVEC, and gene annotations. Bar graphs beneath each track display normalized expression of the corresponding lncRNA target genes in control versus EndMT HUVECs.

In order to explore the gene regulatory impact of the RNA–DNA interactions called for these candidates in endothelial cells, each lncRNA was subjected to siRNA-mediated knockdown in HUVECs followed by RNA-sequencing (Fig. 5a). For each lncRNA, the siRNA-mediated depletion was effective (Supplementary Fig. S2b), although *MIR222HG* did display a transcript-specific depletion. Subsequently, *RADIAnT*-called lncRNA–DNA interactions were intersected with gene loci, permitting statistical enrichment between RNA–DNA interactions and DEG loci to be computed (Fig. 5b). Each of *GAS5, MIR222HG*, and *NUTM2B-AS1* exhibited a degree of enrichment in DNA binding at genes differentially expressed following their knockdowns (*P* = .00032, *P* = .076, and *P* = .00033, respectively), indicating a number of genes whose regulation may be partially dependent on the DNA-binding behavior of the lncRNAs. Notably, this enrichment was greater for *RADIAnT* interactions compared to those called using *BaRDIC* (Supplementary Fig. 2c and d), although *BaRDIC* could still detect a significant enrichment for *NUTM2B-AS1*.

Given these lncRNA candidates were initially selected due to being downregulated in their expression and DNA interaction during EndMT, the pathways and ontologies associated with their putative gene targets were investigated for their physiological relevance to endothelial cell biology. Each module of genes identified per lncRNA displayed significant enrichment of several pathways relevant to endothelial cell function, including actin organization, cytoskeleton organization, and Notch signaling (*GAS5*), actin filaments, cell polarity and wound response (*MIR222HG*) and G2/M transition, cell junction organization and extracellular matrix (ECM) interaction (*NUTM2B-AS1*) (Fig. 5c). Genes implicated in these pathways displayed robust changes in gene expression following lncRNA knockdown (Fig. 5d).

To examine whether these putative RNA-gene regulatory relationships were also present in the original experimental context of EndMT, a single-cell RNA-sequencing experiment where HUVEC were induced to undergo EndMT [46] was utilized. Using these data, the expression patterns of putative directly lncRNA-regulated gene modules were assessed between control and EndMT conditions. For both *MIR222HG* and *NUTM2B-AS1*, significant changes in relative expression of their respective gene modules could be observed between control and EndMT conditions (Fig. 5e), matching the decrease in expression of the lncRNAs across EndMT observed in nucRNA-seq. However, putative si*GAS5* downregulated genes did not follow the expected pattern.

To investigate how these lncRNAs might be regulating their target genes, lncRNA–DNA binding sites were intersected with a ChromHMM [48] model learned on epigenomic data from HUVECs [14]. At several genes, lncRNA binding sites (25 kb in size) intersected with associated gene promoter and enhancer regions, indicating that the presence of these lncRNAs at regulatory elements could be directly responsible for the observed alterations in gene expression observed in isolated (siRNA-mediated) and physiological (EndMT-mediated) perturbation of lncRNA expression (Fig. 5f). Interestingly, in *MIR222HG* knockdown, both genes whose regulatory elements intersected with the called RNA–DNA binding site (*IL1RL1* and *IL18R1*) were differentially expressed following the depletion of the lncRNA. In contrast, *AFMID*, whose locus intersected with a *NUTM2B-AS1* binding site but lacked any annotated gene regulatory elements in that region, was not differentially expressed following *NUTM2B-AS1* knockdown, whereas *BIRC5* was. This demonstrates that with the current resolutions of RNA–DNA data, other epigenomic data is helpful to enhance the precision of assignment of RNA gene regulatory targets and, therefore, to understand the contribution of gene regulatory RNAs to dynamic transcriptional responses.

## Discussion

This study was designed to improve the performance and utility of RNA–DNA interaction calling from all-to-all RNA–DNA ligation data, thereby enabling the identification of gene regulatory RNAs and their direct gene targets. While lncRNAs have been proposed as important regulators of gene expression [5, 7], they are often studied at an individual level. The nature of all-to-all RNA–DNA ligation data, combined with effective interaction calling, enables a less biased and more integrative approach to study actions of gene regulatory RNAs in a genome-wide manner.

To enable this, we propose *RADIAnT*, a novel statistical method capable of extracting biologically meaningful signals from diverse RNA–DNA ligation data. We developed *RADIAnT* to account for the major confounding factors in these data while maintaining sensitivity to detect interactions of lowly expressed RNAs. By constructing dataset-specific backgrounds that consider both distance- and genomic position-specific biases, *RADIAnT* adapts to the particular characteristics of each experiment. This flexibility proved essential for detecting consistent RNA-chromatin interactions across different ligation methods and experimental conditions, as demonstrated by concordant *Malat1* binding patterns identified in independent RADICL-seq [19] and GRID-seq [21, 36] datasets. Benchmarking against orthogonal one-to-all datasets [31] showed that RADIAnT consistently outperforms existing methods in both accuracy and computational efficiency, enabling large-scale integrative analyses that were previously impractical. *RADIAnT* is incorporated into a reads-to-interactions pipeline and is generalizable across any RNA–DNA ligation method. Tailored to utilize the input dataset to build a multifaceted statistical background, *RADIAnT* is formulated to be robust to dataset depth and quality. However, it should be recognized that there remain several limiting factors in the generation and analysis of RNA–DNA interaction data, which cannot be completely addressed here.

For one, the highly complex state of RNA–DNA interactions coupled with the relative inefficiency of ligation methods in returning valid read pairs (Fig. 2c) results in extremely sparse output data. In the experiments analyzed here, we observed an average efficiency (total reads in called interactions versus total reads sequenced) of ~10%. The largest loss of informative reads is to rRNA, which often made up half of the total library. Ideally, this would be selected against in the course of the molecular method as in standard RNA-seq [53]; however, the biotinylated state of the linker oligonucleotides used in RNA–DNA ligation methods makes this difficult because rRNA depletion also relies upon biotin-dependent steps.

The problem of data sparsity could be observed in the benchmarking experiment carried out here. We used *Malat1*-DNA interactions called from RAP-DNA [31], which was sequenced to a depth of ~30 million reads. In contrast, the total RADICL-seq or GRID-seq reads involving *Malat1* were ~2 million and 1 million, respectively. In the cases of the *BaRDIC* [24] and Bonetti *et al*. [19] methods, this sparsity resulted in severe under-calling of *Malat1*-DNA interactions. Conversely, *RADIAnT* and the Zhou *et al*. [36] method tended to overcall, although *RADIAnT* did return closer numbers of interactions to the RAP-DNA experiment. Given that *Malat1* is amongst the highest expressed genes in the genome, the problem of data sparsity is likely to be magnified when considering more lowly expressed RNAs. For this reason, we believe that the increased sensitivity displayed by *RADIAnT* is preferable to prioritizing specificity, and subsequently overlooking potentially interesting RNA–DNA interactions.

Among the most interesting RNA–DNA interactions are those with the potential to influence gene regulation. lncRNAs have increasingly been recognized as important regulators of gene expression via specific patterns of genome-wide localization [7]—yet their precise gene regulatory contributions to dynamic processes remain incompletely understood. In this study, we report putative gene regulatory roles of chromatin-associated lncRNAs in EndMT, a process fundamental to embryonic vascular development, but also implicated in various pathological contexts such as fibrosis, atherosclerosis, and pulmonary hypertension [51]. The interactions called by *RADIAnT* from RADICL-seq data across EndMT show considerable dynamics in lncRNA–DNA contacts between control and EndMT conditions, including long-range interactions with regions of accessible chromatin likely to be gene regulatory regions.

How gene regulatory RNA behavior is precisely controlled remains an open question. However, the data presented here demonstrate that RNA expression and chromatin association are, as would be expected, closely linked. This was shown by integration of RNA-sequencing data with RADICL-seq data, permitting a comparison between nuclear RNA abundance and chromatin association. While many lncRNAs displayed a pattern where their expression changes across EndMT reflected alterations in their total DNA interactions, there were some exceptions. For instance, *TSPOAP1-AS1* (and several other genes that map around the extreme positive section of the *y*-axis in Fig. 4g) exhibited strong changes in chromatin association during EndMT but little to no changes in RNA expression levels. Such cases in which chromatin binding and RNA expression appear uncoupled point to complex regulatory hierarchies. Several potential mechanisms could be at play, including changes in the availability of target DNA sites due to chromatin remodeling, alterations in the RNA-binding proteome between conditions, or even context-specific changes in RNA conformation that might affect DNA-binding [54].

In a case study to demonstrate how interactions called by *RADIAnT* can be utilized to identify gene regulatory RNA targets, we focused on lncRNAs that showed a decrease in both expression and chromatin association during EndMT, namely *GAS5, MIR222HG*, and *NUTM2B-AS1*. In doing so, we uncovered potentially RNA-regulated gene programs, which may contribute to maintaining endothelial cell identity. LncRNA knockdown experiments confirmed that these lncRNAs bind at and regulate genes whose associated pathways are functionally important to endothelial cell behavior. Putative target genes of *GAS5* were involved in RHO GTPase signaling, actin organization, and Notch signaling–processes that are fundamental to endothelial cell morphology and function [55–57]. *MIR222HG* targets included genes controlling cell polarity, actin filament dynamics, and wound response, while genes bound and regulated by *NUTM2B-AS1* regulated cell cycle progression, extracellular matrix interactions and adherens junctions. Importantly, these lncRNA-regulated gene modules showed corresponding co-expression behavior with the respective lncRNAs in single-cell RNA-sequencing data from EndMT [46], confirming their physiological relevance. Our findings suggest that chromatin-associated lncRNAs contribute to the regulation of gene programs that define cellular phenotypes.

While our analyses suggest associations between lncRNA binding and transcriptional changes, statements about causality remain challenging. RNA–DNA proximity alone does not inform us about the underlying mechanism of action. This is best exemplified by the case of *AFMID* and *BIRC5*, whose loci both overlap with a *NUTM2B-AS1* binding site (see Fig. 5f). While *AFMID* lacks annotated regulatory elements at this site, both promoter and enhancer of *BIRC5* intersect the *NUTM2B-AS1* binding site. Consistent with this, *BIRC5*, but not *AFMID*, shows significant changes in expression after *NUTM2B-AS1* depletion. On the other hand, we also observed cases in which gene expression changed following lncRNA knockdown despite the absence of direct RNA binding at the affected locus. These observations demonstrate that spatial proximity alone does not allow for statements about regulatory function. The mechanisms of action remain to be described–lncRNAs might engage in triplex formation with the DNA double helix [9], recruit chromatin modifying proteins [14], modulate enhancer-promoter interactions [58], or influence transcript processing and stability. Indirect effects should also be considered, i.e. a lncRNA might regulate transcription factors that subsequently affect downstream targets. Integrating proximity-ligation data with high-resolution epigenomic maps and protein information will be essential to elucidate these mechanisms.

Conducting integrative analyses, such as that shown here, in other contexts can only become more precise as RNA–DNA ligation data become deeper and more efficient. However, as sequencing throughput grows, the user should be aware of potential caveats when integrating the results with other data types. In our comparative analyses of RNA abundance between ligation-based and expression-based datasets (Fig. 1e and f), differing library sizes resulted in a seemingly bimodal correlation pattern. Specifically, genes detected at low counts in ligation-based datasets, which were sequenced to relatively greater depths, often fell below detection in the more shallow expression-based datasets. This phenomenon reflects the technical asymmetry and underscores the need for cautious interpretation of such integrative analyses, especially when library depths are mismatched.

As RNA–DNA ligation methods increase in efficiency and depth, we are confident that the robust and adaptable formulation of *RADIAnT* will remain capable of effectively detecting biologically relevant RNA–DNA interactions by building dataset-specific statistical backgrounds. Additionally, as data sparsity decreases, we envisage the bin size to become a dynamic parameter in *RADIAnT* interaction calling, yielding more refined interaction boundaries in datasets with greater effective sequencing depth. However, as seen in the computational efficiency of *BaRDIC*—which uses more dynamic bin sizes than *RADIAnT*—this can come at a cost in terms of computational performance.

Increased RNA–DNA interaction resolutions will permit more accurate integration of RNA–DNA ligation data with other data types and provide greater insight into the biological consequences of RNA–DNA interactions. We furthermore envisage the optional incorporation of DNA–DNA contact maps into our pipeline, which will reveal how RNA–DNA interactions might be constrained by higher-order chromatin topology, offering critical context for lncRNA-mediated control of gene expression. Such integration will help us understand whether RNAs can be constrained by topologically associating domains (TADs), mediate inter-TAD communication, or bridge distant regulatory hubs in the nuclear landscape [59].

Ultimately, we view *RADIAnT* not only as a computational framework but as a biological discovery tool that can unify diverse RNA–DNA ligation datasets, link them to complementary data, and thus help uncover the spatial and temporal logic of RNA-mediated gene regulation. Its flexible formulation, along with our commitment to its maintenance and development, should make it applicable to developing molecular methods. The incorporation of *RADIAnT* into a Snakemake pipeline minimizes user input and maximizes reproducibility, facilitating its implementation across different biological contexts. We believe that *RADIAnT* has the potential to become a community-standard platform for the unified analysis of a variety of RNA–DNA ligation data as both molecular methods and biological questions evolve.

## Supplementary Material

gkag304_Supplemental_Files

## Data Availability

Data generated for this manuscript are available at NCBI GEO under the accessions GSE273203 (RADICL-seq), GSE273205 (ATAC-seq), GSE273207 (nucRNA-seq), and GSE304781 (total RNA-seq after siRNA-mediated knockdown). Source code is available at https://github.com/si-ze/RADIAnT and via Zenodo under https://doi.org/10.5281/zenodo.17737559.
