# Supplementary material for: Improved RNA–DNA interaction calling suggests RNA-based gene regulation of phenotypic transitions
Source: Nucleic Acids Res. 2026 Jun 8;54(11):gkag304. doi: 10.1093/nar/gkag304 (PMC13244158; doi:10.1093/nar/gkag304)
Supplement: gkag304_Supplemental_Files [file gkag304_supplemental_files.zip › Figure_S1.pdf]

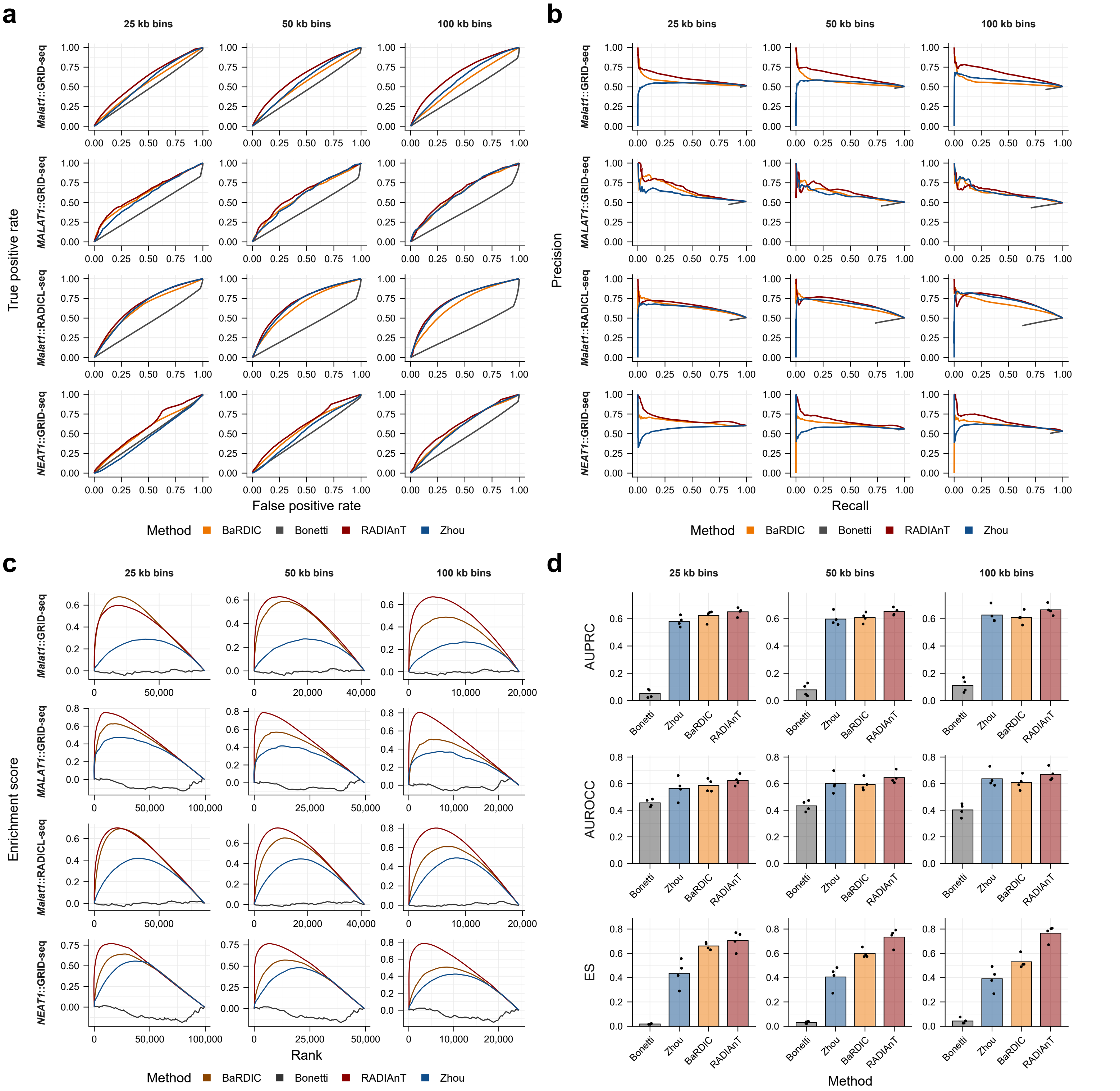

Figure S1. Performance evaluation of RNA-DNA interaction callers using one-to-all RNA-DNA binding data as a ground truth. (a) Receiver operating characteristic (ROC) curves for *BaRDIC*, Bonetti *et al.*, *RADIAnT* and Zhou *et al.* across four evaluation setups and three bin sizes. **(b)** Precision-recall curves for *BaRDIC*, Bonetti *et al.*, *RADIAnT* and Zhou *et al.* across four evaluation setups and three bin sizes. **(c)** Gene set enrichment analysis (GSEA) curves for *BaRDIC*, Bonetti *et al.*, *RADIAnT* and Zhou *et al.* across four evaluation setups and three bin sizes. **(d)** Area under the precision-recall curve (AUPRC), area under the ROC curve (AUROCC) and GSEA enrichment scores (ES) for *BaRDIC*, Bonetti *et al.*, *RADIAnT* and Zhou *et al.* across four evaluation setups (RADICL-seq of murine *Malat1*, GRID-seq of murine *Malat1*, GRID-seq of human *MALAT1*, GRID-seq of human *NEAT1*) and three bin sizes (25 kb, 50 kb, 100 kb).
