# Supplementary material for: Improved RNA–DNA interaction calling suggests RNA-based gene regulation of phenotypic transitions
Source: Nucleic Acids Res. 2026 Jun 8;54(11):gkag304. doi: 10.1093/nar/gkag304 (PMC13244158; doi:10.1093/nar/gkag304)
Supplement: gkag304_Supplemental_Files [file gkag304_supplemental_files.zip › Figure_S2.pdf]

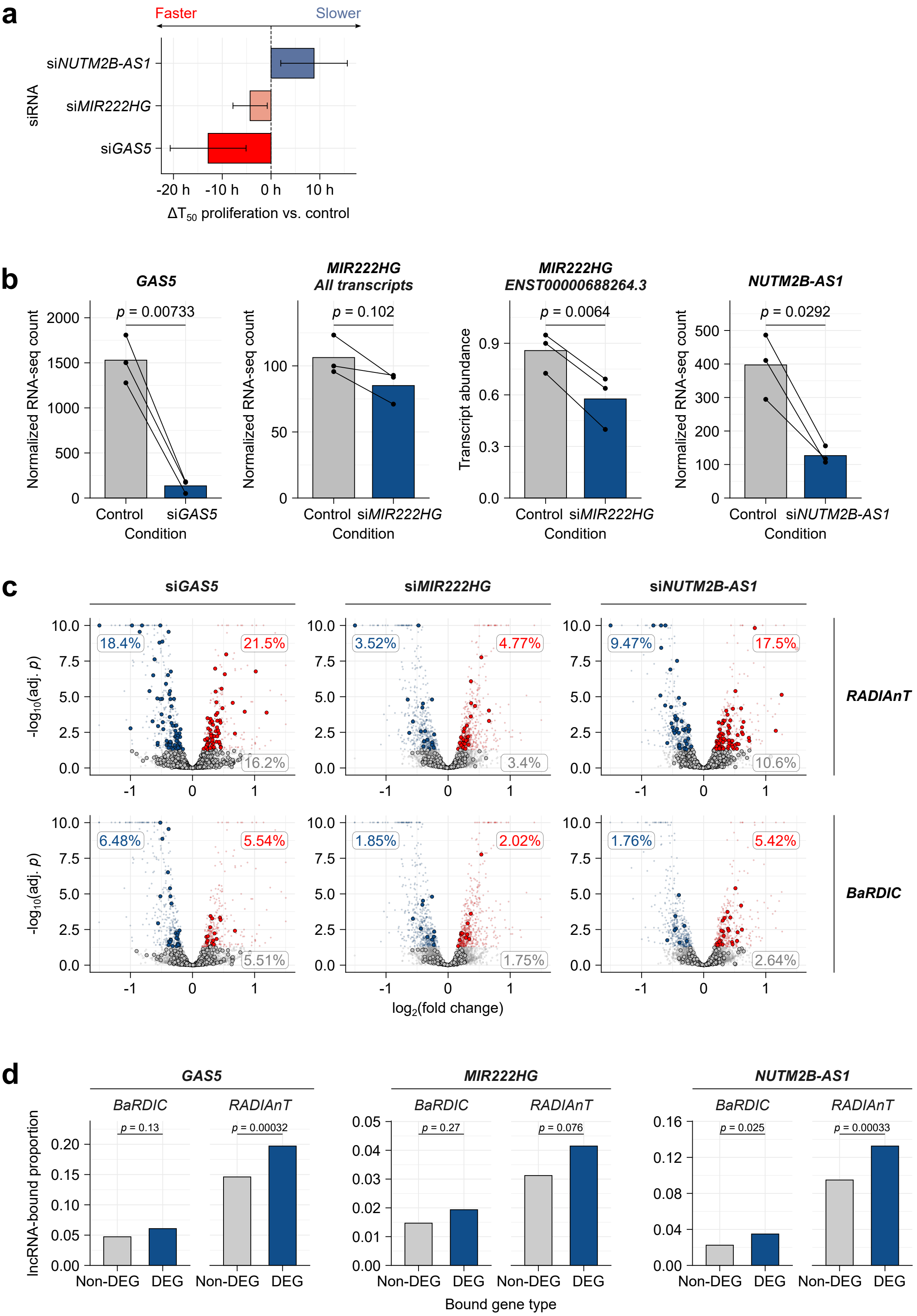

Figure S2. Depletion of candidate lncRNAs is effective and *RADIAnT* detects putative regulatory target genes of lncRNAs more effectively than *BaRDIC*. (a) HUVEC proliferation changes following knockdown of selected lncRNAs, as measured by Incucyte. Data shown represents the change in time taken to reach 50% of maximum confluency compared to control siRNA-treated cells. **(b)** *GAS5*, *MIR222HG* and *NUTM2B-AS1* expression in repsective siRNA-mediated depleted HUVECs, measured by RNA-seq. T-tests were paired by respective donor. **(c)** RNA-sequencing of HUVECs following siRNA-mediated knockdown of *GAS5*, *MIR222HG* and *NUTM2B-AS1*. Highlighted points are those where lncRNA binding was called at the corresponding gene locus from RADICL-seq of HUVECs by *RADIAnT* or *BaRDIC*. Percentages denote the percentage of downregulated, upregulated or non-regulated genes where respective lncRNA binding was called. **(d)** Proportion of differentially expressed genes (DEGs) and non-DEGs where respective lncRNA binding was called by either *RADIAnT* or *BaRDIC*. Non-DEG and DEG proportions were compared using a Chi-square test.
